# Antitumor effects of LPM5140276 and its potential combination with SHP2 inhibition in KRAS^G12D^-mutant cancer

**DOI:** 10.3389/fphar.2025.1554356

**Published:** 2026-01-28

**Authors:** Zhengping Hu, Fangxia Zou, Fengjuan Zhao, Pengfei Yu, Haibo Zhu, Liting Yu, Wenyan Wang, Liang Ye

**Affiliations:** 1 Medicine & Pharmacy Research Center, Binzhou Medical University, Yantai, Shandong, China; 2 School of Pharmacy, Key Laboratory of Molecular Pharmacology and Drug Evaluation (Yantai University), Ministry of Education, Collaborative Innovation Center of Advanced Drug Delivery System and Biotech Drugs in Universities of Shandong, Yantai University, Yantai, Shandong, China; 3 State Key Laboratory of Advanced Drug Delivery and Release Systems, Shandong Luye Pharmaceutical Co., Ltd., Yantai, Shandong, China; 4 School of Pharmacy, Binzhou Medical University, Yantai, Shandong, China; 5 School of Public Health, Binzhou Medical University, Yantai, Shandong, China

**Keywords:** KRAS^G12D^, KRAS^G12D^-mutant cancer, LPM5140276, RMC4550, SHP2 inhibition

## Abstract

KRAS^G12D^ is a predominant mutation in pancreatic and colorectal cancers whose targeting has remained a therapeutic challenge. In this study, we introduced LPM5140276 as a potent KRAS^G12D^ inhibitor that forms a salt bridge with the Asp12 residue; furthermore, we evaluated its antitumor efficacy, action mechanism, and synergy with the SHP2 inhibitor RMC4550. LPM5140276 was observed to bind to GDP-loaded KRAS^G12D^ with high affinity, exhibiting a dissociation constant (K_D_) of 3.1 × 10^−3^ nM and an IC_50_ of 0.5 nM, which was superior to its binding to GTP-loaded KRAS^G12D^. In KRAS^G12D^-mutant cells, LPM5140276 significantly inhibited cell viability by suppressing ERK and AKT phosphorylation to induce G_0_/G_1_ cell-cycle arrest and promote apoptosis, which contributed to its antitumor effect *in vivo*. However, rebound phosphorylation of ERK/AKT and increased SHP2 phosphorylation following the treatment suggested the emergence of bypass resistance. Notably, the combination of LPM5140276 and RMC4550 synergistically suppressed ERK and SHP2 phosphorylation, enhanced G_0_/G_1_ arrest and apoptosis, and improved the antitumor efficacy. Thus, LPM5140276 is a promising KRAS^G12D^ inhibitor, whose combination with SHP2 inhibition represents a viable strategy for overcoming resistance.

## 1 Introduction

Mutations in the RAS family of oncogenes (HRAS, NRAS, and KRAS) are drivers of approximately 20% of all human cancers, with KRAS mutations being the most prevalent type accounting for approximately 80% of these cases. KRAS mutations are particularly common in pancreatic adenocarcinoma (PDAC; 88%), colorectal cancer (CRC; 44%), and lung adenocarcinoma (LUAD; 32%) (Cerami et al., 2012). Among these, the KRAS^G12D^ mutation is the most frequent isoform, which is especially dominant in PDAC (approximately 34% of cases) and also significant in CRC (10%–12%) as well as other malignancies (Li et al., 2018; Hallin et al., 2022). KRAS is a small GTPase with slow intrinsic guanosine triphosphate (GTP) hydrolysis activity that enables switching from the GTP-bound active state to the guanosine diphosphate (GDP)-bound inactive state (Simanshu et al., 2017; Chen et al., 2024). In the GTP-bound state, KRAS activates downstream effector molecules like RAF kinase, which mediates the MAPK pathway, or the PI3K-AKT pathway (Singhal et al., 2024). However, various KRAS mutations can impair GTP hydrolysis or enhance nucleotide exchange to lock KRAS in the GTP-bound active state (Simanshu et al., 2017; Chen et al., 2024).

Despite nearly four decades of research efforts, KRAS was long considered a “non-druggable” target until the discovery of covalent inhibitors targeting the KRAS^G12C^ mutation that exploit a binding pocket in the switch II domain (Ostrem et al., 2013). Numerous covalent inhibitors have been developed since then (Canon et al., 2019; Hallin et al., 2020; Weiss et al., 2022); among these, sotorasib (AMG510) and adagrasib (MRTX849) received accelerated approval by the United States Food and Drug Administration in May 2021 and December 2022, respectively, for the treatment of KRAS^G12C^-mutated non-small-cell lung cancer (NSCLC). However, there are currently no approved drugs for treating KRAS^G12D^ mutations. Promising inhibitors like MRTX1133 have shown potent and specific activities against KRAS^G12D^ cells both *in vitro* and *in vivo* (Wang et al., 2022); furthermore, the phase 1/2 study has been initiated, which will serve as vital proof-of-concept (NCT05737706). Another KRAS^G12D^ inhibitor, namely, HRS-4642, is undergoing phase I/II clinical trials (NCT06427239) (Zhou et al., 2024). Notably, MRTX1133 and HRS-4642 have provided proof-of-principle results for the development of selective non-covalent inhibitors targeting KRAS^G12D^ mutations, confirming the feasibility of the salt-bridge strategy (Wang et al., 2022; Chen et al., 2024; Zhou et al., 2024).

The RAS signaling pathway contains several upstream and downstream mediators that represent attractive targets for combination therapies with RAS inhibitors (Chen et al., 2024; Singhal et al., 2024). In recent years, Src homology-2 domain-containing protein tyrosine phosphatase-2 (SHP2) inhibitors have emerged as promising KRAS-targeted therapies as their inhibition impairs the KRAS nucleotide exchange process (Nichols et al., 2018; Fedele et al., 2021; Liu et al., 2021). The non-receptor protein tyrosine phosphatase SHP2 is encoded by the protein tyrosine phosphatase non-receptor type 11 (*PTPN11*) gene and acts downstream of multiple receptor tyrosine kinases (RTKs) to regulate cell survival and proliferation primarily through activation of the RAS–ERK and PI3K–AKT signaling pathways (Chen et al., 2016). In preclinical models, the SHP2 inhibitors TNO155 and RMC-4630 exhibit synergy with KRAS^G12C^-selective inhibitors (Nichols et al., 2018; Fedele et al., 2021; Liu et al., 2021). In a phase 1/2a study, the combination of glecirasib (KRAS^G12C^ inhibitor) and JAB-3312 (SHP2 inhibitor) achieved an objective response rate (ORR) of 50% and a disease control rate (DCR) of 100% in patients with KRAS^G12C^-inhibitor-naive NSCLC (Wang et al., 2023).

In the present study, we developed a special inhibitor designated as LPM5140276 to target KRAS^G12D^. We observed its inhibitory effects and high selectivity in preclinical models. Furthermore, we found that combining LPM5140276 with the SHP2 allosteric small-molecule inhibitor RMC4550 enhanced the tumor inhibition efficacy in KRAS^G12D^-mutated tumors.

## 2 Materials and methods

### 2.1 Drugs, cells, and chemicals

LPM5140276 and RMC4550 were synthesized by WUXI AppTec Co., Ltd (Nantong, China). The reference molecule MRTX1133 was purchased from MCE Company (United States).

Antibodies against Ras (G12D-mutant specific; #14429), SHP2 (#3752), p-SHP2 (#3751), ERK1/2 (#4695), p-ERK1/2 (#4370), AKT (#4691), p-AKT (#4058), CDK4 (#12790), cyclin D1 (#2922), cleaved caspase-3 (#9661), and cleaved caspase-7 (#9491) were purchased from Cell Signaling Technology. Furthermore, β-tubulin (#66240) was purchased from Proteintech; β-actin (#AA128), penicillin–streptomycin solution (#C0222), and methylthiazolyldiphenyl-tetrazolium bromide (MTT; #ST316) were purchased from Beyotime Biotechnology. RPMI-1640 medium (#11875093), Dulbecco’s modified Eagle medium (DMEM; #11960051), and fetal bovine serum (FBS; #10099141) were purchased from Gibco.

The AsPC-1 (pancreas) and GP2D (colon) tumor cells bearing the KRAS^G12D^ mutation were purchased from the Cell Bank of the Committee on the Type Culture Collection of the Chinese Academy of Sciences (Shanghai, China). The AsPC-1 cells were cultured in RPMI 1640 medium, while the GP2D cells were cultured in DMEM supplemented with 10% FBS and 1% penicillin-streptomycin. All cells were cultured in a humidified incubator at 37 °C with 5% CO_2_.

### 2.2 Synthesis of LPM5140276

**SCHEME 1:**
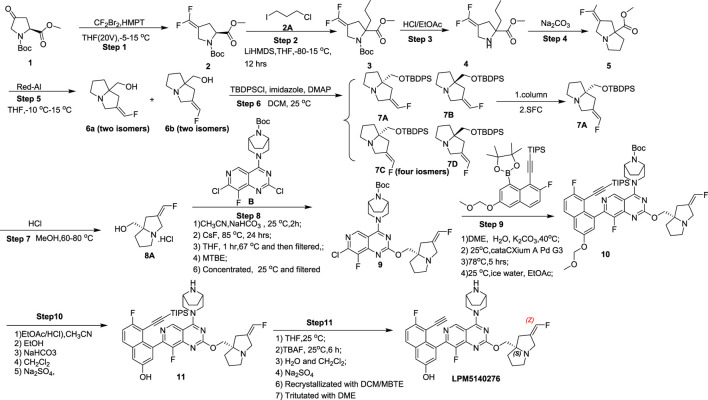
Synthesis route of LPM5140276.

Scheme 1 shows the steps involved in the synthesis of LPM5140276 in this study.

#### 2.2.1 1-(Tert-butyl) 2-methyl (S)-4-(difluoromethylene)pyrrolidine-1,2-dicarboxylate (compound 2)

First, CF_2_Br_2_ (2.2 eq) was added to a solution of compound 1 (1 eq) in tetrahydrofuran (THF; 20 V) at −5 °C; to this solution, we added hexamethylphosphoramide (HMPT; 4.4 eq) dropwise at 0 °C. Then, the mixture was stirred at 15 °C for 14 h. Thin-layer chromatography (TLC) indicated that 10% of compound 1 remained, and one major new spot with lower polarity was detected (petroleum ether/ethyl acetate = 3/1, R_f_ = 0.68). The reaction was poured into an ice–water mixture at 6 V to separate the organic phases. The water phase was extracted with EtOAc once at 7 V and once at 4 V. The combined organic layers were washed with brine at 6 V and concentrated under reduced pressure to obtain a residue. This residue was purified by column chromatography (SiO_2_, petroleum ether/ethyl acetate = 1/0 to 10/1). When the petroleum ether/ethyl acetate ratio was 10/1, the desired compound was obtained as a colorless liquid. ^1^H nuclear magnetic resonance (NMR; 400 MHz, chloroform-*d*) (δ, ppm): 1.35–1.45 (m, 9H), 2.54–2.68 (m, 1H), 2.76–2.98 (m, 1H), 3.68–3.72 (m, 3H), 4.07 (q, *J* = 7.13 Hz, 2H), 4.32–4.55 (m, 1H). ^19^F NMR (376 MHz, chloroform-d) (δ, ppm): −87.7, −87.9, −88.0, −88.1, −90.7, −90.8, −90.8, −90.9.

#### 2.2.2 1-(Tert-butyl) 2-methyl 2-(3-chloropropyl)-4-(difluoromethylene)pyrrolidine-1,2-dicarboxylate (compound 3)

LiHMDS (1.2 eq) was added dropwise to a solution of compound 2 (1 eq) in THF (10 V) at −71 °C, which produced many bubbles. Then, LiHMDS was added further at −66 °C, and the mixture was stirred at −70 °C for 1 h. Next, 1-chloro-3-iodo-propane (1.25 eq) was added dropwise to the solution at −70 °C. The complete reaction was stirred at 15 °C for 12 h. TLC indicated that 0% of compound 1 remained and that one major new spot with a lower polarity could be detected (petroleum ether/ethyl acetate = 3/1, R_f_ = 0.75). To the reaction, we then added a saturated solution of NH_4_Cl at 3.20 V, followed by H_2_O at 1.00 V to separate the organic phase. The organic phase was washed with a saturated solution of NH_4_Cl at 3.20 V. The water phase was combined and extracted at 3.20 V, and the organic phase was also combined. The organic phase was washed with brine at 3.20 V and concentrated at 50 °C under reduced pressure to obtain compound 3. ^1^H NMR (400 MHz, chloroform-*d*) (δ, ppm): 1.37 (d, *J* = 14.0 Hz, 9H), 1.60–1.83 (m, 2H), 1.88–2.04 (m, 2H), 2.11–2.21 (m, 1H), 2.26–2.42 (m, 1H), 2.52–2.84 (m, 2H), 3.41–3.54 (m, 2H), 3.63–3.72 (m, 3H), 4.03–4.23 (m, 2H). ^19^F NMR (377 MHz, chloroform-*d*) (δ, ppm) −91.2, −91.1, −91.0, −90.9, −88.5, −88.3, −88.3, −88.1.

#### 2.2.3 Methyl 2-(3-chloropropyl)-4-(difluoromethylene)pyrrolidine-2-carboxylate (compound 4)

Compound 3 (1 eq) was added to a round-bottom flask along with HCl/EtOAc (4.2 M or 8.49 eq), which produced many bubbles. The reaction was maintained at 15 °C with stirring for 2 h. TLC indicated that 0% of compound 1 remained and that one major new spot with a larger polarity could be detected (petroleum ether/ethyl acetate = 3/1, R_f_ = 0.19). The reaction was adjusted to pH = 9 using Na_2_CO_3_. Then, water was added to the reaction at 2.67 V and 0 °C and stirred for 10 min. The organic phase was filtered and washed with H_2_O at 1.33 V, and the water phase was collected for the next step.

#### 2.2.4 Methyl 2-(difluoromethylene)tetrahydro-1H-pyrrolizine-7a(5H)-carboxylate (compound 5)

Na_2_CO_3_ was added to a solution of compound 4, and the pH was adjusted to 9–10 before being stirred for 2 h. The mixture was extracted with EtOAc thrice at 2.22 V. The combined organic phase was washed with brine at 2.22 V and concentrated at 45–50 °C under a reduced pressure of −0.09 MPa to obtain a residue. This residue was purified by column chromatography (SiO_2_, petroleum ether/ethyl acetate = 1/0 to 10/1); when the petroleum ether/ethyl acetate was 10/1, the desired compound 5 was obtained as a brown oil. ^1^H NMR (400 MHz, chloroform-*d*) (δ, ppm): 1.82–1.94 (m, 3H), 2.33–2.41 (m, 1H), 2.42–2.49 (m, 1H), 2.54–2.65 (m, 1H) 2.96–3.06 (m, 1H), 3.17–3.26 (m, 1H), 3.36 (dd, *J* = 14.4 and 1.2 Hz, 1H), 3.68–3.80 (m, 4H). ^19^F NMR (377 MHz, chloroform-*d*) (δ, ppm): −89.6.

#### 2.2.5 (Z)-(2-(Fluoromethylene)tetrahydro-1H-pyrrolizin-7a(5H)-yl)methanol and (E)-(2-(fluoromethylene)tetrahydro-1H-pyrrolizin-7a(5H)-yl)methanol (compounds 6A and 6B)

Red-Al (70% purity, 4 eq) was added to a solution of compound 5 (1 eq) in THF (10 V) at −10 °C, and this mixture was stirred at 15 °C for 16 h. TLC indicated that 0% of compound 1 remained and that one major new spot with a larger polarity could be detected (petroleum ether/ethyl acetate = 3/1, R_f_ = 0.01). The reaction was cooled to 0 °C and 13.5% NaOH solution was added to the mixture at 3.25 V. When a solid formed, the liquid supernatant was separated; the solid was washed with THF at 3.25 V. The combined organic phase was concentrated at 45–50 °C under reduced pressure of −0.09 MPa to obtain compound 6 in two isomeric forms as a brown oil. ^1^H NMR (400 MHz, chloroform-*d*) (δ, ppm): 1.59–2.00 (m, 5H), 2.18–2.67 (m, 4H), 2.98–3.11 (m, 1H), 3.20–3.34 (m, 4H), 3.36–3.74 (m, 3H), 5.28–5.59 (m, 0.5H), 5.94–6.31 (m, 0.5H), 6.34–6.66 (m, 1H). ^19^F NMR (377 MHz, chloroform-*d*) (δ, ppm): −131.8, −130.7, −114.8.

#### 2.2.6 (S,Z)-7a-(((Tert-butyldiphenylsilyl)oxy)methyl)-2-(fluoromethylene)hexahydro-1H-pyrrolizine (compound 7A)

A mixture of compound 6 (1 eq), tert-butyldiphenylchlorosilane (2 eq), 4-dimethylaminopyridine (0.1 eq), and imidazole (4 eq) in dichloromethane (10 V) was degassed and purged with N_2_ thrice before being stirred at 25 °C for 12 h under N_2_ atmosphere. Liquid chromatography mass spectrometry (LC-MS; EB4756-76-P1A2) showed that 45.7% of the desired compound could be detected. To the reaction mixture, we then added H_2_O at 5.3 V to separate the organic phase. The water phase was extracted with CH_2_Cl_2_ (22 mL), and the organic phases were combined. The combined organic phase was dried over Na_2_SO_4_, filtered, and concentrated under reduced pressure to obtain a residue. Next, 36 mL of methyl tert-butyl ether (MTBE), 71 mL of n-heptane, and 71 mL of HCl (2 M) were added to the crude product to obtain the oil phase. The mixture showed three phases, and oil as the middle phase was separated. The oil phase was washed with MTBE/n-heptane (1:2, 4 V) and dissolved in EtOAc at 8.89 V before washing with Na_2_CO_3_ solution to adjust the pH of the water phase to 9. This water phase was then separated and extracted with EtOAc at 1.33 V. The combined organic phase was washed with brine at 4.44 V and concentrated under reduced pressure to obtain a residue. This residue was purified by column chromatography (SiO_2_, petroleum ether/ethyl acetate = 1/0 to 5.5/1) to obtain the desired compounds 7A and 7B, which were then purified by supercritical fluid chromatography [SFC; column: DAICEL CHIRALCEL OX (250 mm × 50 mm, 10 µm); mobile phase: (0.1% NH_3_H_2_O ETOH); B%: 35%–35%, min] to obtain compound 7A as a yellow oil. ^1^H NMR (400 MHz, chloroform-*d*) (δ, ppm): 1.09 (s, 9H), 1.60–1.70 (m, 1H), 1.71–1.90 (m, 2H), 1.96–2.08 (m, 1H), 2.21 (d, *J* = 15.2 Hz, 1H), 2.47–2.70 (m, 2H), 3.01–3.14 (m, 1H), 3.27–3.50 (m, 3H), 3.75 (d, *J* = 14.8 Hz, 1H), 6.26–6.56 (m, 1H), 7.34–7.47 (m, 6H), 7.68 (dd, *J* = 7.60 and 1.60 Hz, 4H). ^19^F NMR (377 MHz, chloroform-*d*) (δ, ppm): −131.6.

#### 2.2.7 (S,Z)-7a-(((Tert-butyldiphenylsilyl)oxy)methyl)-2-(fluoromethylene)hexahydro-1H-pyrrolizine hydrochloride (compound 8A)

HCl (6 M, 6 V, 15 eq) was added to a solution of compound 7A (1 eq) in MeOH (3 V) at 25 °C, and the resulting mixture was stirred at 80 °C for 12 h. The reaction was filtered, and the filtrate was extracted with MTBE (5.50 V) thrice before concentrating the water phase under reduced pressure to obtain compound 8A as a black solid. ^1^H NMR (400 MHz, methanol-*d*4) (δ, ppm): 1.97–2.14 (m, 2H), 2.17–2.28 (m, 2H), 2.67 (dd, *J* = 15.6 and 1.60 Hz, 1H), 2.85 (dd, *J* = 16.0 and 2.00 Hz, 1H), 3.25 (dt, *J* = 11.8 and 6.69 Hz, 1H), 3.64–3.78 (m, 3H), 4.07 (d, *J* = 14.9 Hz, 1H), 4.29 (d, *J* = 14.9 Hz, 1H), 6.69–7.02 (m, 1H). ^19^F NMR (377 MHz, methanol-*d*4) (δ, ppm): −127.9.

#### 2.2.8 Tert-butyl (1R,5S)-3-(7-chloro-8-fluoro-2-(((S,Z)-2-(fluoromethylene)tetrahydro-1H-pyrrolizin-7a(5H)-yl)methoxy)pyrido[4,3-d]pyrimidin-4-yl)-3,8-diazabicyclo[3.2.1]octane-8-carboxylate (compound 9)

Na_2_SO_4_ (3.00 kg) was added to a solution of compound 8A (3 kg, 12.68 mol, 87.8% purity by QNMR, 1 eq, HCl) in CH_3_CN (45 L) and dried under N_2_ to obtain solution 1. To this solution 1, we then added compound B (5.43 kg, 12.68 mol, 1 eq) and NaHCO_3_ (2.13 kg, 25.36 mol, 2 eq) and stirred the mixture for 2 h at 25 °C under N_2_ atmosphere; then, CsF (963.05 g, 6.34 mol, 0.5 eq) was added, and the mixture was stirred at 85 °C for 24 h. LC-MS (EB8246-79-P1A7) showed that 0% of compound 8A remained. We next added 30.0 L of THF to this reaction and stirred at 67 °C for 1 h. The reaction was filtered and concentrated under reduced pressure to obtain 9.00 L of the solution; this solution was added to 9.00 L of MTBE and concentrated under reduced pressure to obtain 9.00 L of the resultant solution, which was then cooled to 25 °C and filtered to obtain compound 9 (3.5 kg, 6.03 mol, 47.5% yield, 97.9% purity, QNMR 97.4% assay) as a white solid. ^1^H NMR (400 MHz, CDCl_3_) (δ, ppm): 1.49 (s, 9H), 1.63–1.78 (m, 3H), 1.87–1.97 (m, 4H), 2.07–2.16 (m, 1H), 2.32 (d, *J* = 15.6 Hz, 1H), 2.58–2.76 (m, 2H), 3.14–3.21 (m, 1H), 3.40 (d, *J* = 14.8 Hz, 1H), 3.64 (s, 2H), 3.86 (d, *J* = 14.8 Hz, 1H), 4.05–4.24 (m, 2H), 4.27–4.56 (m, 4H), 6.48 (d, *J* = 84.8 Hz, 1H), 8.70 (s, 1H). ^19^F NMR (400 MHz, CDCl_3_) (δ, ppm): −130, −134.

#### 2.2.9 Tert-butyl (1R,5S)-3-(8-fluoro-7-(7-fluoro-3-(methoxymethoxy)-8-((triisopropylsilyl)ethynyl)naphthalen-1-yl)-2-(((S,Z)-2-(fluoromethylene)tetrahydro-1H-pyrrolizin-7a(5H)-yl)methoxy)pyrido[4,3-d]pyrimidin-4-yl)-3,8-diazabicyclo[3.2.1]octane-8-carboxylate (compound 10)

Compound 9A (2.17 kg, 4.24 mol, 1.40 eq) and K_2_CO_3_ (1.26 kg, 9.08 mol, 3.00 eq) was added to a solution of compound 9 (1.75 kg, 3.03 mol, 97.4% purity by QNMR, 1.00 eq) in dimethoxyethane (DME; 35 L) and H_2_O (3.5 L) with stirring at 25 °C under N_2_ atmosphere; the mixture further stirred for 30 min at 40 °C to obtain a clear solution. To this, we added cataCXium A Pd G_3_ (110.24 g, 151.37 mmol, 0.05 eq) at 25 °C, and the mixture was degassed and purged with N_2_ thrice before stirring at 78 °C for 6 h. LC-MS and high-performance liquid chromatography (HPLC) showed that 0% of compound 9 remained at this stage. The reaction mixture was quenched with 8.80 L of H_2_O (0 °C) and 8.80 L of EtOAc solution at 20–30 °C before separating the organic phase. The water phase was extracted with 8.80 L of EtOAc. The combined organic layers were washed with 10.2 L of brine, dried over Na_2_SO_4_, and concentrated under reduced pressure to obtain a residue. The crude product was next purified using SiO_2_ (12.0 kg) and petroleum ether/ethyl acetate at 5/1 (40 L) to remove the impurities, followed by additional purification with petroleum ether/ethyl acetate at 5/1 (50 L) to obtain the desired compound. The two-part desired compound solution was concentrated under reduced pressure to obtain compound 10 (4.64 kg, 4.45 mol, 73.4% yield, 93.0% purity, QNMR 87.5% assay) as a brown solid. ^1^H NMR (400 MHz, CDCl_3_) (δ, ppm): 0.47–0.61 (m, 3H), 0.88 (dd, *J* = 7.38 and 4.75 Hz, 19H), 1.24–1.31 (m, 12H), 1.54 (s, 11H), 1.66–1.87 (m, 4H), 1.93–2.04 (m, 7H), 2.14–2.28 (m, 1H), 2.39 (d, *J* = 15.2 Hz, 1H), 2.62–2.85 (m, 2H), 3.26 (br s, 1H), 3.39–3.58 (m, 6H), 3.76–3.99 (m, 2H), 4.16–4.26 (m, 3H), 4.32–4.93 (m, 4H), 5.18–5.45 (m, 2H), 6.31–6.75 (m, 1H), 7.28–7.31 (m, 1H), 7.32 (d, *J* = 2.80 Hz, 1H), 7.52 (d, *J* = 2.40 Hz, 1H), 7.79 (dd, *J* = 9.20 and 5.60 Hz, 1H), 9.06 (s, 1H). ^19^F NMR (400 MHz, CDCl_3_) (δ, ppm): −106, −131, −137.

#### 2.2.10 4-(4-((1R,5S)-3,8-Diazabicyclo[3.2.1]octan-3-yl)-8-fluoro-2-(((S,Z)-2-(fluoromethylene)tetrahydro-1H-pyrrolizin-7a(5H)-yl)methoxy)pyrido[4,3-d]pyrimidin-7-yl)-6-fluoro-5-((triisopropylsilyl)ethynyl)naphthalen-2-ol (compound 11)

HCl/EtOAc (4.5 M, 16.24 L, 16.44 eq) was added to a solution of compound 10 (4.64 kg, 4.45 mol, 1.00 eq) in acetonitrile (9.3 L) and EtOAc (8.1 L) at 25 °C under N_2_ atmosphere, and the mixture was stirred at 25 °C for 1 h until a solid formed appeared. LC-MS and HPLC showed that 0% of compound 10 remained. The reaction was filtered and washed with EtOAc (2.30 L) under N_2_; the filter cake was then dissolved in 18.6 L of EtOAc and added to a solution of NaHCO_3_ (1.87 kg, 2.513 mol, 5.00 eq, in 18.6 L of H_2_O, 0 °C) at 0–10 °C. When the pH was equal to 8, we added 18.6 L of CH_2_Cl_2_ to the solution and separated the water phase. The water phase was extracted with CH_2_Cl_2_ (13.9 L). The combined organic layers were washed with brine (37.1 L), dried over Na_2_SO_4_, filtered, and concentrated under reduced pressure to obtain compound 11 (4.15 kg, 4.35 mol, 97.8% yield, 95.0% purity, QNMR 80.6% assay) as a brown solid. ^1^H NMR (400 MHz, dimethylsulfoxide-*d*_6_) (δ, ppm): 0.45 (quin, *J* = 8.00 Hz, 3H), 0.80 (t, *J* = 7.60 Hz, 18H), 1.60–1.71 (m, 2H), 1.77–2.00 (m, 6H), 2.34 (d, *J* = 15.6 Hz, 1H), 2.51–2.64 (m, 2H), 2.95–3.07 (m, 1H), 3.29 (d, *J* = 14.4 Hz, 1H), 3.42–3.52 (m, 1H), 3.66–3.88 (m, 5H), 3.94–4.09 (m, 2H), 4.19 (br d, *J* = 12.0 Hz, 1H), 4.70 (d, *J* = 12.8 Hz, 1H), 6.725 (d, *J* = 85.6 Hz, 1H), 7.17 (d, *J* = 2.00 Hz, 1H), 7.39 (d, *J* = 2.40 Hz, 1H), 7.46 (t, *J* = 8.80 Hz, 1H), 7.96 (dd, *J* = 9.20 and 6.00 Hz, 1H), 9.11 (s, 1H). ^19^F NMR (400 MHz, CDCl_3_) (δ, ppm): −109, −131, −140.

#### 2.2.11 4-(4-((1R,5S)-3,8-Diazabicyclo[3.2.1]octan-3-yl)-8-fluoro-2-(((S,Z)-2-(fluoromethylene)tetrahydro-1H-pyrrolizin-7a(5H)-yl)methoxy)pyrido[4,3-d]pyrimidin-7-yl)-5-ethynyl-6-fluoronaphthalen-2-ol (compound LPM5140276)

Tetrabutylammonium fluoride (1 M, 6.52 L, 1.5 eq) was added to a solution of compound 11 (4.15 kg, 4.35 mol, 1 eq) in THF (14.2 L) at 25 °C under N_2_ atmosphere, and the mixture was stirred at 25 °C for 6 h. LC-MS and HPLC showed that 0% of compound 11 remained. The reaction was poured into a mixture containing 21.0 L of H_2_O and 10.4 L of CH_2_Cl_2_ to separate the organic phase, following which the water phase was extracted with CH_2_Cl_2_ (8.30 L) twice. The combined organic layers were washed with 14.0 L of H_2_O, dried over Na_2_SO_4_, filtered, and concentrated under reduced pressure to obtain a crude product. This crude product was dissolved in 3.00 L of CH_2_Cl_2_ followed by dropwise addition of MTBE (6.00 L) and was cooled to 25 °C to obtain a solid. The solid was treated with DME (30.0 L) twice to obtain the final compound LPM5140276 (1.16 kg, 1.84 mol, 42.3% yield, 97.1% purity) as a pale yellow solid. ^1^H NMR (400 MHz, dimethylsulfoxide-*d*_6_) (δ, ppm): 1.60–2.01 (m, 8H), 2.31 (d, *J* = 14.8 Hz, 1H), 2.52–2.61 (m, 2H), 2.95–3.03 (m, 1H), 3.28 (d, *J* = 14.8 Hz, 2H), 3.47–3.59 (m, 3H), 3.60–3.75 (m, 2H), 3.94 (s, 1H), 3.98–4.11 (m, 2H), 4.30 (d, *J* = 12.0 Hz, 1H), 4.47 (d, *J* = 11.2 Hz, 1H), 6.74 (d, *J* = 85.6 Hz, 1H), 7.17 (d, *J* = 2.40 Hz, 1H), 7.39 (d, *J* = 2.40 Hz, 1H), 7.46 (t, *J* = 8.80 Hz, 1H), 7.97 (dd, *J* = 9.20 and 6.00 Hz, 1H), 9.04 (s, 1H). ^19^F NMR (400 MHz, dimethylsulfoxide-*d*_6_) (δ, ppm): −111, −131, −140.

### 2.3 Molecular docking studies

Three docking programs (CDOCKER, Glide XP, and GOLD) were evaluated preliminarily, and Glide extra precision (XP) was ultimately selected owing to its superior performance in root mean-squared deviation (RMSD) testing. The redocking study demonstrated that Glide could reproduce the bioactive crystal conformation of MRTX1133 with an RMSD value of 0.1247 Å (Glide XP mode).

#### 2.3.1 Protein structure preparation

The X-ray structure of the KRAS^G12D^ protein in complex with MRTX1133 (PDB code: 7RPZ) was retrieved from the Protein Data Bank (PDB) for docking-based studies. All water molecules were removed from this structure, and the missing bond orders were edited. Then, hydrogen atoms were added, and the complex structure was submitted to Schrödinger for protein preparation. The module automatically optimizes the states of hydroxyl, Asn, Gln, and His residues using ProtAssign, followed by restrained minimization with the OPLS_2005 force field. The refined structure was then used for grid calculations to define a binding site with a size similar to the bound ligand.

#### 2.3.2 Ligand preparation

LPM5140276 was sketched in ChemBioDraw, converted into different 3D structures, and subjected to local minimization using the OPLS_2005 force field. The resulting structures were used for subsequent docking simulations.

#### 2.3.3 Docking procedure

The docking studies were performed using Glide XP mode with flexible ligand sampling. Flips of 5- and 6-member rings were allowed, while non-polar conformations of any amide bonds were penalized. At most, 10 positions were investigated per ligand, while all other parameters were retained with default values. Finally, the 10 best conformations were saved for further analyses.

### 2.4 Surface plasmon resonance (SPR)

The binding of LPM5140276 and MRTX1133 to the KRAS^G12D^ enzyme was determined at 25 °C using the Biacore 8K SPR system (GE Healthcare) by WuXi AppTec (Shanghai, China). Here, Avi-KRAS^G12D^ (corresponding to amino acids 1:169; 1 μg/mL) was immobilized on a Serial-S CM5 Sensorchip (GE Healthcare) via the classic amine-coupling method in an immobilization buffer containing 10 mM of HEPES, 150 mM of NaCl, 0.5 mM of TCEP, and 0.05% (v/v) of the surfactant P20. Single-cycle kinetic measurements were performed in a running buffer containing 50 mM of Tris (pH 8.8), 50 mM of NaCl, 0.5 mM of TCEP, 5 mM of MgCl_2_, 0.05% of the surfactant P20, and 2% dimethylsulfoxide (DMSO). The compounds were diluted in a threefold series with the running buffer, where the DMSO concentration was carefully adjusted to 2%. The compound solutions were injected into the prepared sensor chips at a flow rate of 80 μL/min for 80 s and allowed to dissociate over approximately 5,000 s. The data were analyzed using Biacore 8K evaluation software with the 1:1 kinetic binding model.

### 2.5 Active and inactive KRAS^G12D^ assays

We performed biochemical binding assays for inactive KRAS (GDP-loaded) and RBD binding assays for active KRAS (GMP-PNP-loaded) with LPM5140276.

The binding ability to inactive wild-type KRAS (KRAS^WT^) or KRAS^G12D^ was assessed using a time-resolved fluorescence resonance energy transfer (TR-FRET) displacement assay. In 10 µL of the assay, we added 5 µL of biotinylated KRAS^WT^ or KRAS^G12D^ (amino acids 1–169 from Accelegan) to ECHO650 (Beckman Coulter) dispensed compound dissolved in DMSO along with 5 μL of Cy5-labeled tracer and terbium streptavidin (Cisbio). The final assay conditions included 10 nM of Cy5-labeled tracer, 0.5 nM of terbium streptavidin, and the compound (with 1% DMSO) in the buffer (50 mM of HEPES, pH 7.5, 5 mM of MgCl_2_, 0.005% Tween 20, and 1 mM of dithiothreitol). Following 1 h of incubation at room temperature, the reactions were measured using a BMG CLARIOstar Plus device via TR-FRET. The IC_50_ values were fitted to a four-parameter IC_50_ equation using XLfit software (IDBS). One hundred percent of control (POC) was determined using DMSO as the control, and 0 POC was achieved using a certain concentration of the control compound that completely inhibited tracer binding to KRAS.

For active KRAS (GMP-PNP loaded) measurement, biotinylated KRAS^WT^ or KRAS^G12D^ was combined with GST-tagged Raf1-RBD (Cell Signaling Technology) and incubated with 2.5 nM of anti-GST-d2 (Cisbio) and 0.5 nM of terbium streptavidin in 10 µL of the assay containing predispersed compounds dissolved in DMSO. After 1 h of incubation at room temperature, the reactions were measured using BMG CLARIOstar Plus via TR-FRET. The IC_50_ values were calculated, and 0 POC was determined using a concentration of the control compound that completely inhibited binding of RAF-RBD to KRAS.

### 2.6 Cell viability assays

Cell viability was evaluated using the MTT assay as described previously. In brief, AsPC-1 and GP2D cells were seeded in 96-well microplates at a density of 6,000 cells per well and cultured overnight in complete growth media. The cancer cells were treated with various concentrations of the compounds (LPM5140276 with and without RMC4550) for an additional 48 h, whereas the control cells were treated with DMSO (final concentration = 0.1%). Then, the cell viability was measured at OD_570nm_ using a Molecular Devices SpectraMax M5 (Sunnyvale, CA, United States) as per the manufacturer’s protocols. All data are the averages of at least three independent experiments. The online SynergyFinder Plus software (https://www.synergyfinderplus.org) was used to calculate the drug synergy scores using the inhibition index and zero interaction potency (ZIP) calculation method. Accordingly, ZIP synergy scores greater than 0 were considered synergistic (red regions), and scores greater than 10 were considered strongly synergistic. Heatmaps of the drug combination responses were also generated to assess the therapeutic significances of the combinations.

### 2.7 Western blotting

The AsPC-1 cells were seeded in 10-cm-diameter dishes at a density of 2 × 10^6^ cells per dish. Upon reaching 60%–70% confluence, the cells were treated with LPM5140276 either alone or in combination with RMC4550. The culture medium was aspirated at specified timepoints, and the cells were rinsed with ice-cold phosphate-buffered saline (PBS) for 30 min prior to lysis on ice with RIPA buffer supplemented with phenylmethanesulfonyl fluoride (PMSF) and phosphatase inhibitor. The lysates were centrifuged at 12,000 rpm for 30 min, and the protein concentrations were quantified with Pierce BCA protein assay kits (Thermo Fisher Scientific, United States). The protein samples were separated with 6%–15% SDS-PAGE gel and transferred to polyvinylidene fluoride membranes. The membranes were blocked in 5% non-fat milk solution for 2 h at room temperature and incubated overnight at 4 °C with primary antibodies against Ras (G12D-mutant specific) (1:1,000), ERK1/2 (1:1,000), p-ERK1/2 (1:1,000), AKT (1:1,000), p-AKT (1:1,000), SHP2 (1:1,000), p-SHP2 (1:1,000), CDK4 (1:1,000), cyclin D1 (1:1,000), cleaved caspase-3 (1:1,000), cleaved caspase-7 (1:1,000), and tubulin (1:1,000). On the following day, the membranes were incubated with horseradish peroxidase (HRP)-conjugated secondary antibodies (1:5,000) for 2 h at room temperature. The chemiluminescent signals were detected using an enhanced chemiluminescence kit.

### 2.8 Cell-cycle and apoptosis analyses by flow cytometry

For the cell-cycle analysis, the AsPC-1 and GP2D cells were seeded in 6-well plates at a density of 2 × 10^5^ cells per well, allowed to adhere overnight, and treated with various concentrations of LPM5140276 and/or RMC4550 for 24 h or 48 h. The treated cells were harvested and washed thrice with PBS. Each sample was fixed in 500 μL of ice-cold 80% ethanol at 4 °C for 2 h or overnight, rinsed thrice with PBS, and incubated with 500 μL of RNase A/propidium iodide (PI) mixture (1:9, KeyGEN BioTECH, China) for 30–60 min at room temperature in the dark. The cell-cycle distribution was analyzed using a flow cytometer (BD, United States).

For the apoptosis analysis, the AsPC-1 and GP2D cells were seeded in 6-well plates at a density of 1 × 10^5^ cells per well overnight and treated with LPM5140276 and/or RMC4550 for 24 h or 48 h. The cells were collected by centrifugation at 800*g* for 5 min, washed twice with PBS, and resuspended in 1× binding buffer at a concentration of 1 × 10^6^ cells/mL. Next, approximately 100 μL of this solution was stained with 5 μL of Annexin V-FITC and 5 μL of PI (BD, United States), gently vortexed, and incubated for 15 min at room temperature in the dark. The apoptosis was then analyzed using a flow cytometer.

### 2.9 Pharmacokinetics in mice

LPM5140276 was dissolved with 10% captisol in 50 mM of citrate buffer (pH 5.0) to achieve concentrations of 0.1, 0.3, and 1 mg/mL. Then, male mice received intraperitoneal injections of LPM5140276 at doses of 1, 3, 10 mg/kg (n = 4 per group). Venous blood samples were collected from the mice prior to dosing and at multiple timepoints post-administration; these samples were placed in heparinized tubes and centrifuged at 8,000 rpm for 10 min at 4 °C to separate the plasma, which was then stored at −20 °C until analysis. The plasma samples were pretreated by liquid–liquid extraction with a mixture of n-hexane, dichloromethane, and isopropanol (2:1:0.1, v/v/v) for quantification and validation by liquid chromatography tandem mass spectrometry (Thermo Electron Corporation, CA, United States). The pharmacokinetic parameters were calculated by non-compartmental analysis using WinNonlin software (version 6.3, Pharsight Corporation, CA, United States).

### 2.10 Antitumor efficacies in the KRAS^G12D^-mutant xenograft models

Male Balb/c nu/nu nude mice (5–6 weeks old; Vital River Laboratory Animal Technology Co., Ltd.) were used for the *in vivo* experiments. These animals were housed in laminar-flow cabinets under controlled conditions at 25 °C with 12-h light/dark cycles as well as *ad libitum* access to food and water. All animal protocols were approved by the Ethics Committee of Binzhou Medical University (No. 20221014–22 for Animal Ethics Approval), and efforts were made to minimize animal suffering. The tumor xenograft models (six animals/group) were established by subcutaneous injections of 3 × 10^6^ AsPC-1 or GP2D cells into the right flanks of each of the animals. Treatments were initiated when the tumors volumes reached 100–140 mm^3^. LPM5140276 was administrated intraperitoneally once daily for 21 d or 28 d at doses of 1, 3, or 10 mg/kg. The tumor dimensions and body weights of the animals were recorded twice weekly. The tumor volume was calculated using the following formula: volume (mm^3^) = 0.5 × length (mm) × width (mm) × height (mm).

### 2.11 Statistical analysis

The data were analyzed by two-tailed Student’s t-test and ANOVA using GraphPad Prism (GraphPad Software Inc., San Diego, CA, United States). The results are presented as mean ± standard deviation (SD) or standard error of the mean (SEM), and *p* values ≤0.05 were considered as statistically significant.

## 3 Results

### 3.1 Docking analysis of potential binding modes of LPM5140276

**FIGURE 1:**
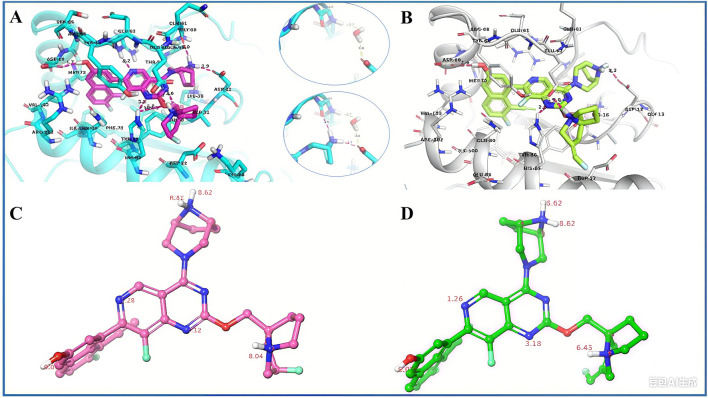
**(A)** X-ray crystal structure of MRTX1133 in a complex with KRAS^G12D^/GDP (PDB code: 7RT4). **(B)** Optimal docking conformation of LPM5140276 with KRAS^G12D^. **(C)** pKa values of the individual nitrogen atoms in MRTX1133. **(D)** pKa values of the individual nitrogen atoms in LPM5140276.

To investigate the binding modes of LPM5140276 in the reported allosteric pocket of KRAS^G12D^ (MRTX1133 site, PDB code: 7RPZ), optimal molecular docking (Glide XP, Schrödinger) was performed (Figure 1; Supplementary Table S1). As depicted in Figure 1, the positively charged hexahydro-1H-pyrrolizine of LPM5140276 and pyrrolidinyl moiety of MRTX1133 at the C2 position were all extended toward the solvent region to form a strong salt bridge with Glu62. Additionally, the C7-naphthyl of both compounds occupies a deep hydrophobic pocket, where the 3-hydroxyl substituent of the naphthyl ring forms hydrogen bonds with Asp69. Given the importance of the salt bridge with Asp12 to the activity and selectivity of the MRTX1133 and LPM5140276 molecules, we found that the distances of the terminal hydrogen atoms of the amine moiety and Asp12 were 2.3 Å and 2.2 Å, respectively. These three vectors were identified as opportunities to increase the affinity of the KRAS^G12D^ protein.

### 3.2 SPR K_D_ determination

SPR was used to determine the kinetics and affinity between LPM5140276 or MRTX1133 and the KRAS^G12D^ proteins. The SPR signal intensity is correlated with the concentration of LPM5140276 or MRTX1133 bound to the KRAS proteins immobilized on the sensor chips, where a lower equilibrium dissociation constant (K_D_) indicates greater affinity. SPR confirmed the specific bindings of both compounds to KRAS^G12D^, with K_D_ values of 3.1 × 10^−3^ nM for LPM5140276 and 1.8 × 10^−3^ nM for MRTX1133 (Table 1).

**TABLE 1:** Direct binding of LPM5140276 and MRTX1133 to KRAS^G12D^ as measured by surface plasmon resonance.

Compound	K_D_ (nM)	Ka (M^–1^s^–1^)	Kd (s^−1^)
MRTX1133	1.8 × 10^−3^	2.3 × 10^7^	4.2 × 10^−5^
LPM5140276	3.1 × 10^−3^	1.7 × 10^7^	5.3 × 10^−5^

### 3.3 KRAS^G12D^ active- and inactive-state binding assays

**FIGURE 2:**
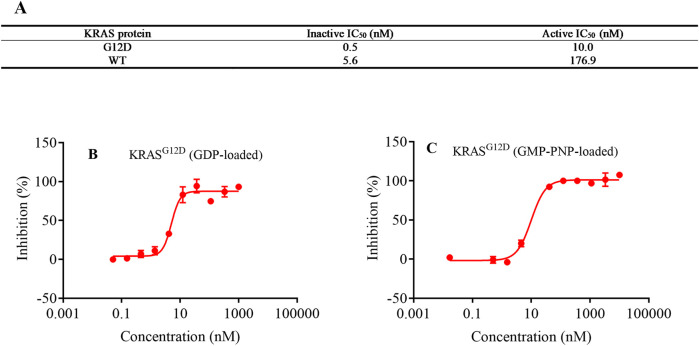
**(A)** IC_50_ values for LPM5140276 bound to KRAS^G12D^ and KRAS^WT^ were determined using **(B)** inactive KRAS (GDP-loaded) biochemical binding assay and **(C)** active KRAS (GMP-PNP-loaded) RBD binding assay (n = 2).

To characterize the binding preferences of LPM5140276 to the active and inactive states of KRAS^G12D^ and KRAS^WT^, the IC_50_ values were determined using biochemical binding assays with inactive KRAS (GDP-loaded) and RBD binding assays with active KRAS (GMP-PNP-loaded) (Figure 2). The results demonstrated that LPM5140276 could bind to both active and inactive states of KRAS^G12D^ and KRAS^WT^. For KRAS^G12D^, the IC_50_ values were 0.5 nM (inactive) and 10.0 nM (active); in contrast, LPM5140276 exhibited significantly lower affinity for KRAS^WT^, with IC_50_ values of 5.6 nM (inactive) and 176.9 nM (active).

### 3.4 SHP2 inhibitor RMC4550 synergizes with LPM5140276 to suppress tumor cell viability

**FIGURE 3:**
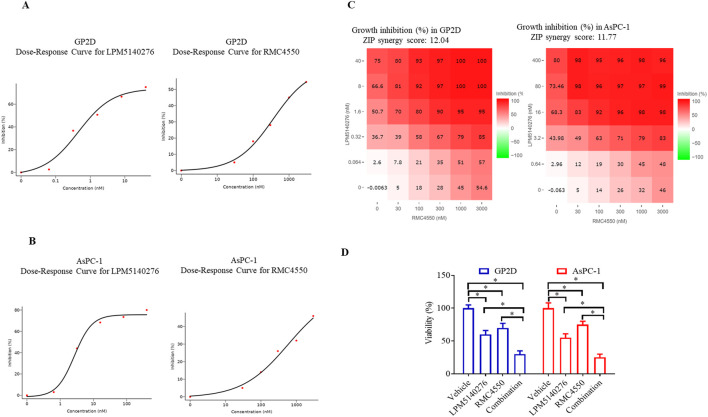
Combination of LPM5140276 and RMC4550 exerts synergistic antiproliferative effects on tumor cells. **(A, B)** Dose–response curves of LPM5140276 and RMC4550 alone, showing the growth inhibition matrices of LPM5140276 in combination with RMC4550 in **(A)** GP2D or **(B)** AsPC-1 cell lines. **(C)** Heatmaps of the drug combination responses. LPM5140276 and RMC4550 exhibit synergistic effects on GP2D and AsPC-1 cells. The cells were treated with LPM5140276 and RMC4550 at the indicated concentrations for 48 h, and the cell viability was assessed via MTT assay. The zero interaction potency (ZIP) synergy scores were calculated using SynergyFinder software, where scores >0 indicate synergism and scores >10 denote strong synergism. **(D)** Cell viabilities of GP2D and AsPC-1 cells treated with vehicle, LPM5140276, RMC4550 (300 nM), and their combination for 48 h. The LPM5140276 concentrations used were 0.32 nM for the GP2D and 3.2 nM for the AsPC-1 cells. The data are presented as mean ± standard error of the mean (SEM) (n = 3) and were analyzed by ANOVA followed by Tukey’s test; *p < 0.05.

The MTT assay was used to evaluate the antitumor activities in the AsPC-1 and GP2D tumor cells following 48 h of treatment with various concentrations of LPM5140276 alone or in combination with RMC4550. The results indicate that RMC4550 synergistically enhances the antiproliferation activities of LPM5140276 against both cell lines. As shown in Figures 3A,B, LPM5140276 and RMC4550 each exhibited concentration-dependent antiproliferative effects. The IC_50_ values of LPM5140276 against GP2D and AsPC-1 tumor cells were 0.3 nM and 3.8 nM, while those of RMC4550 were 1,787 nM and >3,000 nM, respectively. Using the online SynergyFinder software, the ZIP synergy scores were calculated from dose–response matrices, which revealed average interaction contributions of 12.04 in the GP2D cells and 11.77 in the AsPC-1 cells for the overall antitumor responses (Figure 3C). Indeed, treatment with LPM5140276 and RMC4550 showed highly synergistic effects at inhibiting tumor proliferation (ZIP synergy scores >10). Further analysis showed that 300 nM of RMC4550 was the minimum concentration for the highest synergy in both cell lines and was thus selected as the optimal combination concentration. For LPM5140276, concentrations of 0.32 nM (GP2D) and 3.2 nM (AsPC-1) were determined to maximize the synergistic effects.

As shown in Figure 3D, treatment with LPM5140276 at 0.32 nM significantly inhibited the growth of GP2D cells (*p* < 0.05, n = 3) compared to the control group; RMC4550 at 300 nM also decreased the cell growth significantly (*p* < 0.05, n = 3). Furthermore, we observed that the combination of LPM5140276 and RMC4550 was more potent than LPM5140276 or RMC4550 alone in reducing the growth of the tumor cells (*p* < 0.05 each, n = 3); the inhibition rates were 40%, 30%, and 70% for the LPM5140276, RMC4550, and combination groups, respectively.

Similarly, LPM5140276 at 3.2 nM significantly inhibited the growth of AsPC-1 cells (*p* < 0.05, n = 3) compared to the control group; RMC4550 at 300 nM also decreased the cell growth significantly (*p* < 0.05, n = 3). Furthermore, the combination of LPM5140276 and RMC4550 was more potent than LPM5140276 or RMC4550 alone in reducing the growth of the tumor cells (*p* < 0.05 each, n = 3); here, the inhibition rates were 45%, 25%, and 75% for the LPM5140276, RMC4550, and combination groups, respectively (Figure 3D).

### 3.5 LPM5140276 and RMC4550 combination induces cell-cycle arrest and cell apoptosis

**FIGURE 4:**
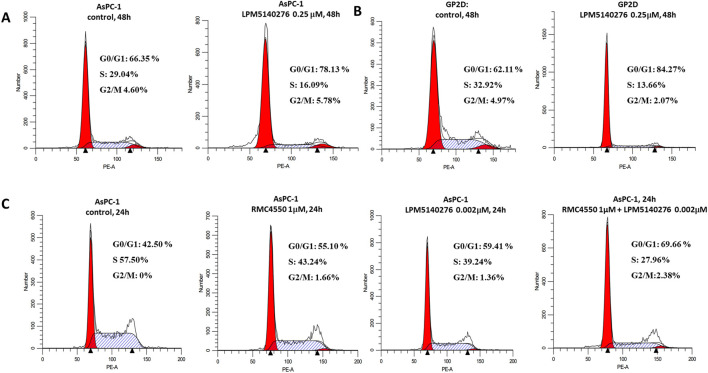
Combination of LPM5140276 and RMC4550 induces cell-cycle arrest. **(A, B)** LPM5140276 treatment led to significant increases in the proportions of cells in the G_0_/G_1_ phase and decreases in S-phase cells in both the AsPC-1 and GP2D lines. **(C)** RMC4550 monotherapy increased G_0_/G_1_-phase arrest in the AsPC-1 cells at 24 h. Compared with either agent individually or the control, the combination of LPM5140276 and RMC4550 resulted in a significantly higher proportion of cells arrested in the G_0_/G_1_ phase as well as a marked reduction in S-phase cells at 24 h.

Flow cytometry analysis was used to evaluate the cell-cycle effects of LPM5140276 and/or RMC4550 in the AsPC-1 and GP2D cells. As shown in Figure 4, LPM5140276 treatment altered cell-cycle distribution to increase the proportions of cells in the G_0_/G_1_ phase and decrease S-phase populations in both cell lines (Figures 4A,B); RMC4550 alone also induced G_0_/G_1_ arrest in AsPC-1 cells at 24 h (Figure 4C). Notably, the combination treatment with LPM5140276 and RMC4550 resulted in significantly greater G_0_/G_1_-phase accumulation and S-phase reduction than single-agent treatments or controls at 24 h (Figure 4C).

**FIGURE 5:**
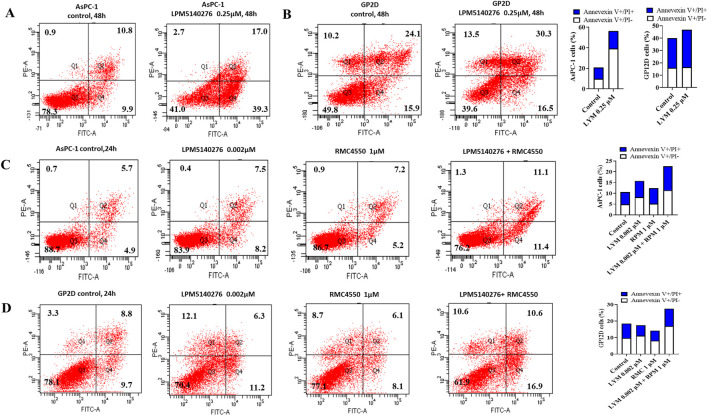
Combination of LPM5140276 and RMC4550 induces cell apoptosis. **(A, B)** LPM5140276 monotherapy induced significant increases in the apoptosis rates of AsPC-1 and GP2D cells. **(C, D)** RMC4550 treatment caused modest elevations in apoptosis. At 24 h, the combination of LPM5140276 and RMC4550 induced significantly higher apoptosis in both AsPC-1 and GP2D cells than either agent singly or the control.

Parallel Annexin V-FITC/PI staining assays were used to evaluate the apoptotic effects following 24 h or 48 h of treatment. Here, LPM5140276 induced significant apoptosis in both cell lines at 48 h (Figures 5A,B), while RMC4550 alone caused a modest apoptotic increase in AsPC-1 cells. Importantly, the combination treatment triggered significantly higher apoptosis rates in both AsPC-1 and GP2D cells compared to single-agent treatments or controls at 24 h (Figures 5C,D).

### 3.6 Effects of LPM5140276 and/or RMC4550 on the signaling pathways related to proliferation, cell-cycle arrest, and apoptosis

**FIGURE 6:**
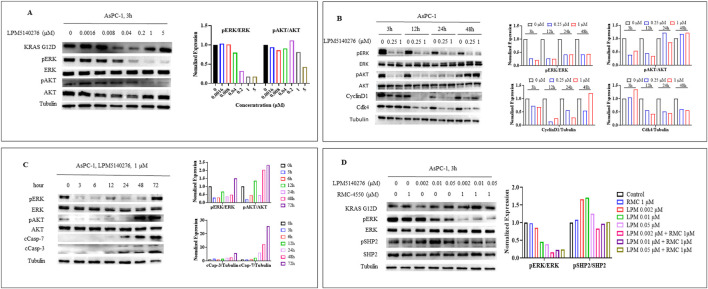
Effects of LPM5140276 and/or RMC4550 on the signaling pathways related to cell proliferation, cell-cycle arrest, and apoptosis. **(A)** In the AsPC-1 cells, p-ERK and p-AKT levels were reduced in a dose-dependent manner 3 h after LPM5140276 treatment. **(B)** LPM5140276-treated AsPC-1 cells showed downregulated CDK4 and cyclin D1 expression at 3 h, 12 h, 24 h, and 48 h. **(C)** p-ERK levels decreased from 3 h to 48 h but rebounded at 72 h, while p-AKT levels decreased from 3 h to 24 h and rebounded at 48 h. LPM5140276 treatment upregulated cleaved caspase-3 and cleaved caspase-7 levels from 3 h to 72 h in the AsPC-1 cells. **(D)** LPM5140276 monotherapy reduced p-ERK level in a concentration-dependent manner but increased p-SHP2 level in the AsPC-1 cells at 3 h. Compared to single-agent treatments, combined administration of RMC4550 and LPM5140276 at increasing concentrations resulted in greater suppression of both p-ERK and p-SHP2 at 3 h.

Western blotting analysis was performed to investigate the effects of LPM5140276 and/or RMC4550 on the critical signaling pathways in KRAS^G12D^-mutant AsPC-1 cells. Compared with the control group, treatment with LPM5140276 resulted in dose-dependent reductions in p-ERK and p-AKT levels in the AsPC-1 cells after 3 h (Figure 6A). Furthermore, the p-ERK levels continued to decrease from 3 h to 48 h but rebounded at 72 h (Figure 6C), whereas the p-AKT levels continued to decrease from 3 h to 24 h and rebounded at 48 h (Figure 6C). These findings confirm that LPM5140276 inhibits the activation of both MEK/ERK and PI3K/AKT/mTOR signaling pathways.

Further analysis showed that LPM5140276 treatment reduced the p-ERK levels in AsPC-1 cells along with decreased CDK4 and cyclin D1 expression at 3, 12, 24, and 48 h, indicating that LPM5140276 induces G_0_/G_1_-phase cell-cycle arrest (Figure 6B). Additionally, LPM5140276 treatment induced time-dependent increases in the apoptosis markers, including cleaved caspase-3 and cleaved caspase-7, in the AsPC-1 cells from 3 h to 72 h (Figure 6C).

Since SHP2 is a key mediator of RAS/ERK signaling, we examined the single-agent effects of the SHP2 inhibitor RMC4550 in the AsPC-1 cells. Notably, no significant suppression of p-ERK was observed following 3 h of treatment with RMC4550 (1 μM) alone. In contrast, LPM5140276 monotherapy suppressed p-ERK in a concentration-dependent manner at 3 h while increasing the p-SHP2 level in the AsPC-1 cells (Figure 6D). Compared with the individual agents, the combination of RMC4550 and LPM5140276 at increasing concentrations resulted in greater suppression of both p-ERK and p-SHP2 at 3 h, confirming that this combination prevents MAPK signaling activation (Figure 6D).

### 3.7 Pharmacokinetics in mice

**FIGURE 7:**
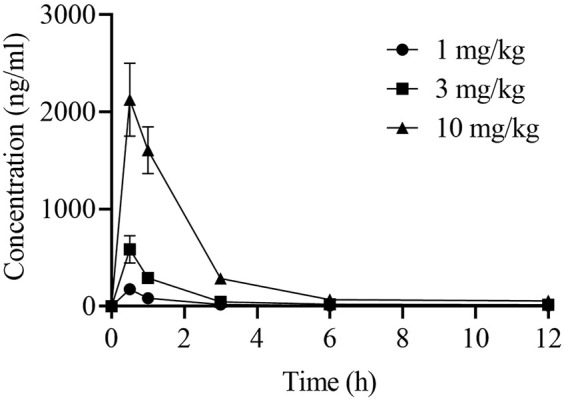
Pharmacokinetic profile of LPM5140276 showing the mean plasma LPM5140276 concentration–time curve in mice administered LPM5140276 at 1, 3, and 10 mg/kg intraperitoneally.

To characterize the pharmacokinetic properties of LPM5140276 in mice, the plasma concentrations were measured using high-performance liquid chromatography tandem mass spectrometry. For the dose range of 1–10 mg/kg, the mean C_max_ and AUC_last_ of LPM5140276 generally increased in a dose-dependent manner; the half-life (t_1/2_) values were 3.91, 3.57, and 2.51 h for dose concentrations of 1, 3, and 10 mg/kg, respectively (Table 2; Figure 7).

**TABLE 2:** Pharmacokinetic findings in mice.

Group	t_1/2_ (h)	T_max_ (h)	C_max_ (ng/mL)	AUC_last_ (h·ng/mL)	AUC_last_/dose (h·ng/mL)/(mg/kg)	MRT_last_ (h)
1 mg/kg	3.91	0.5	176.33	298.63	298.63	2.69
3 mg/kg	3.57	0.5	586.67	914.86	304.95	2.19
10 mg/kg	2.51	0.5	2,126.67	4,269.46	426.95	2.01

### 3.8 Antitumor efficacies in the KRAS^G12D^-mutant xenograft models

**FIGURE 8:**
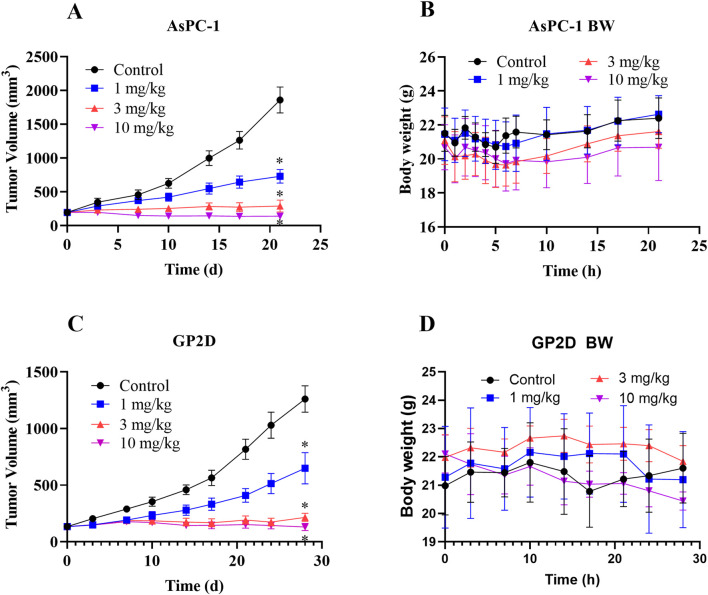
Antitumor effects of LPM5140276 *in vivo* on nude mice subcutaneously inoculated with AsPC-1 or GP2D cells. **(A, C)** LPM5140276 at 1, 3, and 10 mg/kg/d significantly reduced the tumor volumes in a dose-dependent manner. The data are presented as mean ± standard deviation (SD) (n = 6). and were analyzed by ANOVA followed by Tukey’s test; *p < 0.05 versus control. **(B, D)** LPM5140276 did not cause obvious weight losses in the two models.

The *in vivo* efficacy of LPM5140276 was evaluated using two xenograft models, i.e., Balb/c nu/nu nude mice implanted with AsPC-1 and GP2D cells subcutaneously. In both models, LPM5140276 at doses of 1, 3, and 10 mg/kg/d reduced tumor volumes significantly (day 21, *p* < 0.05 each, n = 6) compared to vehicle controls at the end of the study, with inhibition rates of 60.7%, 84.4%, and 92.5% in the AsPC-1 model as well as 48.7%, 82.9%, and 97.6% in the GP2D model, respectively (Figures 8A,C). Importantly, intraperitoneal administration of LPM5140276 did not cause significant weight loss, suggesting favorable safety profiles in both models (Figures 8B,D).

## 4 Discussion

RAS genes represent the most frequently mutated oncogenes in human cancers, with the mutant RAS proteins acting as key drivers in some of the deadliest malignancies, including most pancreatic cancers, half of the colorectal cancers, and one-third of lung cancers (Simanshu et al., 2017; Singhal et al., 2024). Among them, KRAS^G12D^ is a particularly potent target for the design and development of inhibitors in human cancers (Hallin et al., 2022).

In this study, we successfully developed LPM5140276 as a KRAS^G12D^ inhibitor using a salt-bridge-based design strategy. Docking analyses of the potential binding modes of LPM5140276 revealed distinct characteristics compared to MRTX1133, while SPR assay demonstrated comparable binding affinity with KRAS^G12D^. Notably, LPM5140276 bound to the inactive state of KRAS^G12D^ with 20-fold higher affinity than to the active state. Conversely, the compound exhibited significantly lower affinities with KRAS^WT^ in both conformational states.

Sustained KRAS signaling activates pro-tumorigenic pathways, including the Ras/Raf/MEK/ERK and PI3K/AKT/mTOR cascades, which play critical roles in promoting cell proliferation and survival to enhance angiogenesis, facilitate metastasis, and reprogram cellular metabolism to meet the energy demands of the rapidly dividing cancer cells (Ying et al., 2012; Commisso et al., 2013; Dey et al., 2020). In our experiments, LPM5140276 displayed concentration-dependent antiproliferative effects against AsPC-1 and GP2D tumor cells, which are likely attributable to the reduced phosphorylations of ERK (p-ERK) and AKT (p-AKT).

Western blotting analysis of LPM5140276-treated AsPC-1 cells revealed decreased CDK4 and cyclin D1 expression, indicating G_0_/G_1_-phase cell-cycle arrest. Flow cytometry corroborated these findings, showing increased G_0_/G_1_-phase populations and corresponding decreases in S-phase cells in both the AsPC-1 and GP2D cultures. The flow cytometry assessments also demonstrated significantly elevated apoptosis rates in LPM5140276-treated cells, which was confirmed by Western blotting showing increased levels of cleaved caspase-3 and cleaved caspase-7 as established apoptosis markers.

Furthermore, *in vivo* studies demonstrated favorable pharmacokinetic properties of LPM5140276 in mice. In nude mouse models bearing AsPC-1 or GP2D xenografts, LPM5140276 produced dose-dependent reductions in the tumor volume, consistent with its *in vitro* antiproliferative activity, cell-cycle arrest, and apoptotic effects. Importantly, LPM5140276 exhibited good tolerability in both models, with no significant observable weight loss, suggesting minimal off-target effects.

The efficacies of pathway-targeted anticancer agents are frequently limited by drug resistance mediated through bypass signaling pathway activation (Ahmed et al., 2019), as observed with KRAS^G12C^ inhibitors. Rapid adaptive resistance to KRAS^G12C^ inhibitors has been linked with the activation of multiple RTKs (Ryan et al., 2020; Yaeger and Solit, 2020). SHP2 is a critical component in different oncogenic signaling pathways, including RAS/RAF/MAPK and PI3K/AKT/mTOR signaling (Ruess et al., 2018; Torres-Ayuso and Brognard, 2018; Hao et al., 2019; Shen et al., 2020). SHP2 inhibitors like RMC4550 or SHP2 gene knockout can block RAS/MAPK signaling to inhibit RTK-driven tumor growth (Chen et al., 2016; Nichols et al., 2018). Additionally, targeted therapies can induce RTK/SHP2-mediated bypass resistance in tumors driven by KRAS mutations, ALK rearrangements, and other oncogenic alterations (Mainardi et al., 2018; Nichols et al., 2018; Ruess et al., 2018; Ahmed et al., 2019; Lu et al., 2019). As a convergent node in the bypass resistance mechanisms (Ruess et al., 2018), SHP2 inhibition combined with appropriate targeted agents can synergistically reduce tumor RAS-GTP levels while suppressing RAF/MEK/ERK and PI3K/AKT/mTOR signaling to overcome the bypass resistance and enhance the antitumor effects (Nichols et al., 2018; Ryan et al., 2020; Yaeger and Solit, 2020). Preclinical models and clinical trials have also demonstrated such synergy between SHP2 inhibitors and KRAS^G12C^-selective inhibitors (Nichols et al., 2018; Ryan et al., 2020; Yaeger and Solit, 2020; Fedele et al., 2021; Liu et al., 2021).

Time-dependent feedback reactivation of the KRAS–ERK pathway has been observed with the KRAS^G12D^ inhibitor MRTX1133 in tumor cells and models, potentially limiting its efficacy (Hallin et al., 2022). In the study, researchers identified SHP2 inhibition as a co-therapeutic strategy with the potential to augment MRTX1133 responses, consistent with observations from KRAS^G12C^ inhibitor studies (Hallin et al., 2022). Further pharmacological investigations showed that combining SHP2 inhibition with MRTX1133 to target putative feedback and resistance pathways significantly improved the antitumor efficacies in pancreatic and colorectal cancers (Hallin et al., 2022).

In our study, the treatment of AsPC-1 cells with LPM5140276 resulted in p-ERK rebound at 72 h and p-AKT rebound at 48 h, suggesting that feedback reactivation of these pathways may limit the efficacy of LPM5140276. Interestingly, while LPM5140276 suppressed p-ERK levels in AsPC-1 cells at 3 h in a concentration-dependent manner, it concurrently increased p-SHP2 levels, suggesting that SHP2 may represent a key bypass resistance node leading to subsequent ERK and AKT reactivation. Further studies confirmed that co-treatment with RMC4550 (SHP2 inhibitor) and LPM5140276 produced greater suppression of both p-ERK and p-SHP2 at 3 h, indicating that this combination may prevent MAPK pathway reactivation. Importantly, the combination treatment with LPM5140276 and RMC4550 resulted in increased G_0_/G_1_-phase cell population and enhanced apoptosis compared to either agent alone, suggesting synergistic effects on cell-cycle arrest and apoptosis. SynergyFinder software analysis confirmed highly synergistic antiproliferative effects with this combination, which was validated in both the GP2D and AsPC-1 cell models.

In summary, we successfully developed LPM5140276 as a novel KRAS^G12D^ inhibitor. This compound exhibits potent antitumor activities *in vitro* and *in vivo*, which are mediated through reduced p-ERK and p-AKT levels, G_0_/G_1_-phase cell-cycle arrest, and induction of apoptosis. The *in vitro* studies demonstrate that SHP2 inhibition combined with LPM5140276 produces synergistic effects on tumor cell proliferation reduction, cell-cycle arrest, and apoptosis. These findings suggest that combining SHP2 inhibitors with LPM5140276 may address the bypass resistance pathways to significantly improve the antitumor efficacies of these drugs.

## Data Availability

The original contributions presented in the study are included in the article/Supplementary Material, and any further inquiries may be directed to the corresponding author.
