# Supplementary material for: Antitumor effects of LPM5140276 and its potential combination with SHP2 inhibition in KRASG12D-mutant cancer
Source: Front Pharmacol. 2026 Jan 28;16:1554356. doi: 10.3389/fphar.2025.1554356 (PMC12891233; doi:10.3389/fphar.2025.1554356)

Table S1 Computational parameters of docking procedures and other physicochemical properties

| ID | Global strain (Kcal/mol) | Glide GScore | PKa (Nitrogen atom in Solvent region) |
| --- | --- | --- | --- |
| MRTX-1133 | 5.512 | -10.733 | 8.04 |
| LPM5140276 | 5.681 | -11.219 | 6.43 |

Fig. S1 The raw sensorgrams. A for MRTX1133 and B for LPM5140276.


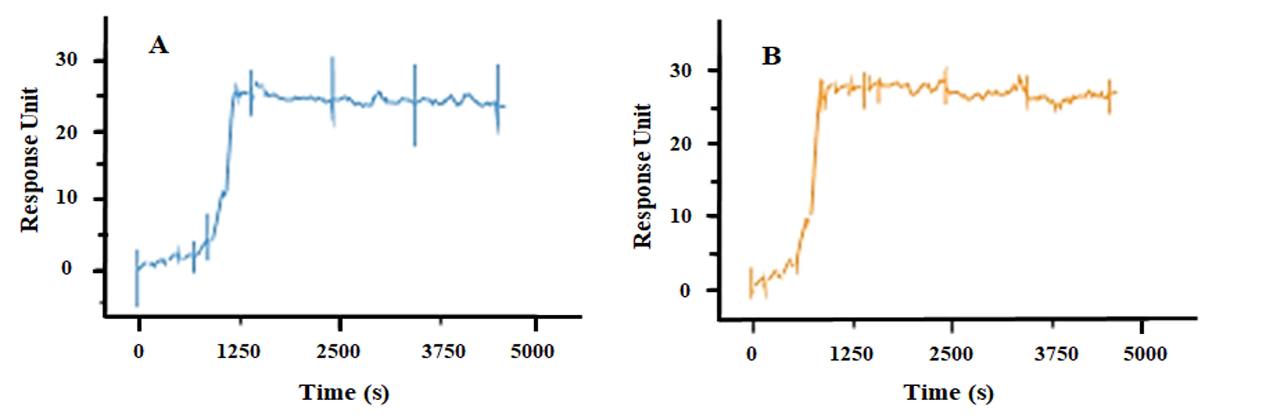


Fig S2. The representative FACS plots for cell cycle arrest.


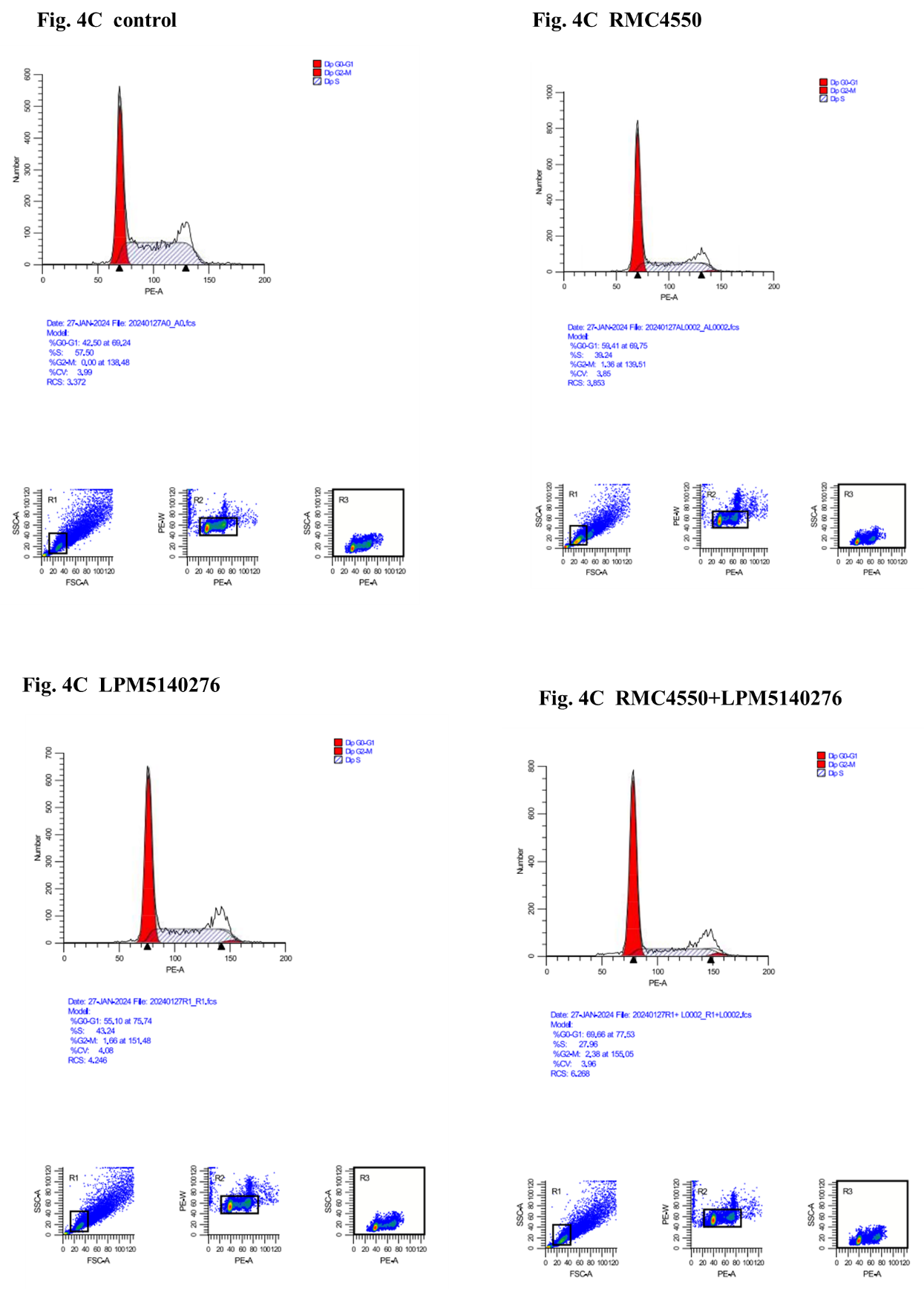


Fig S3. The representative FACS plots for cell apoptosis


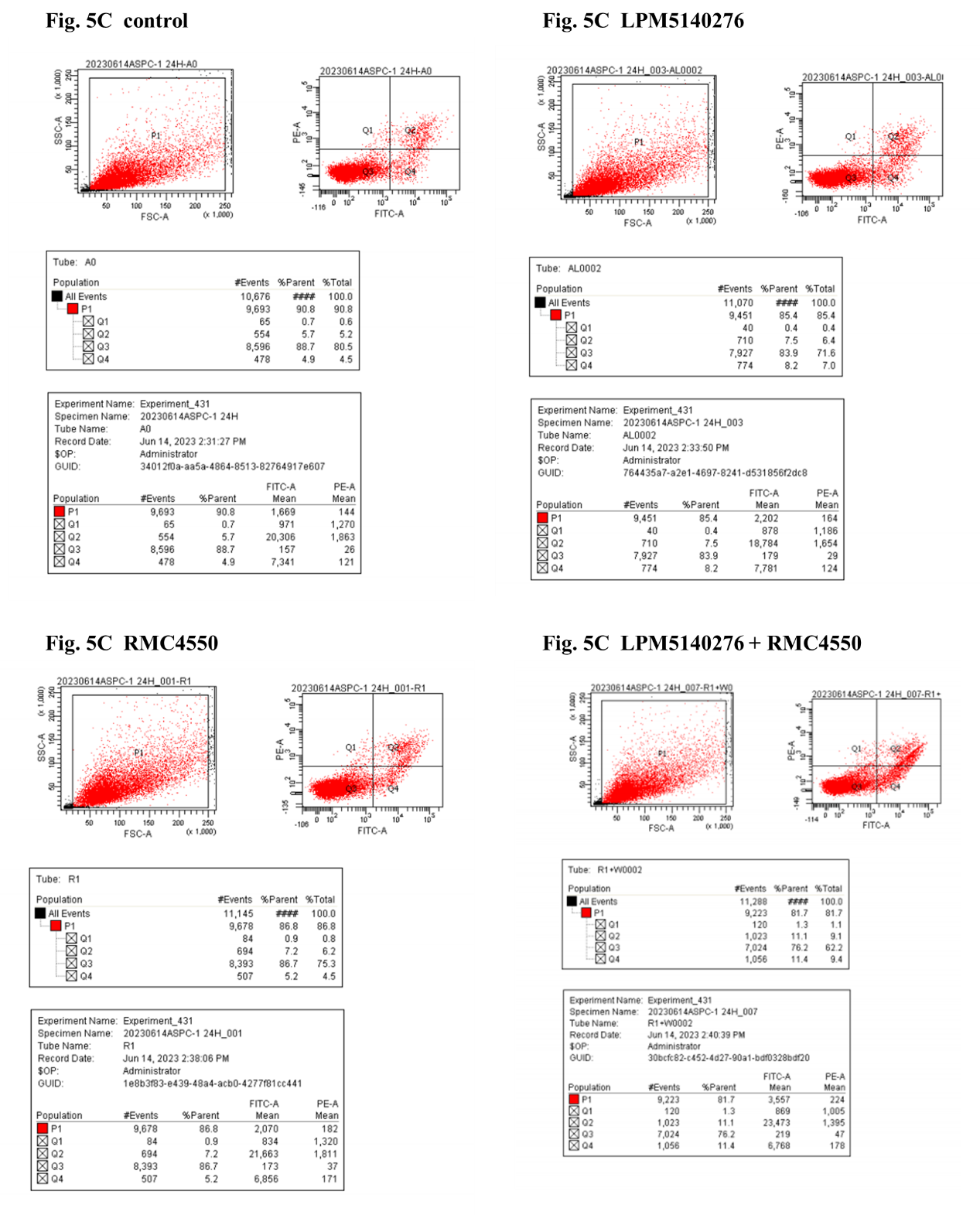


Fig S4. The uncropped blots for Fig. 6.

Fig. 6A

Kras G12D
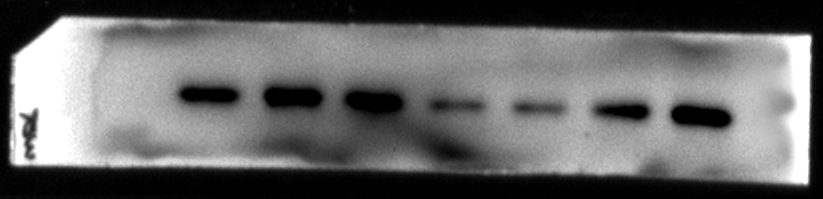


pERK
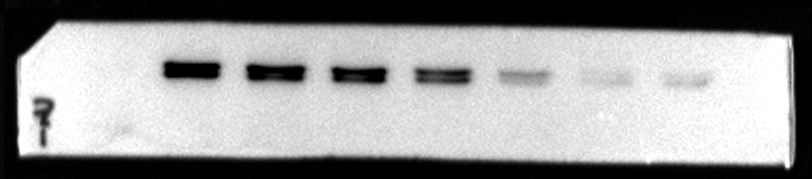


ERK
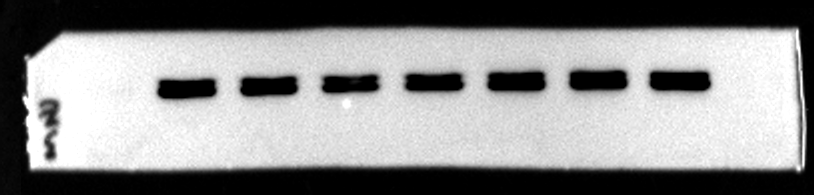


pAKT
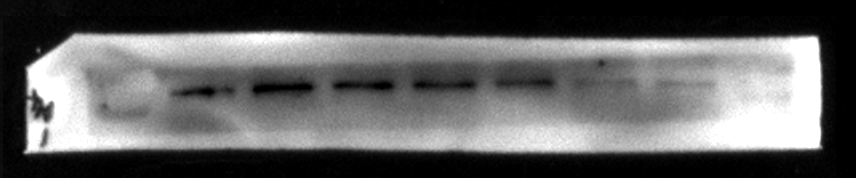


AKT
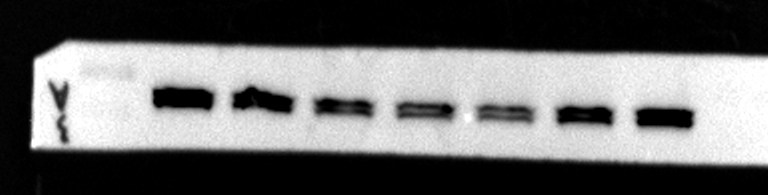


Tubulin
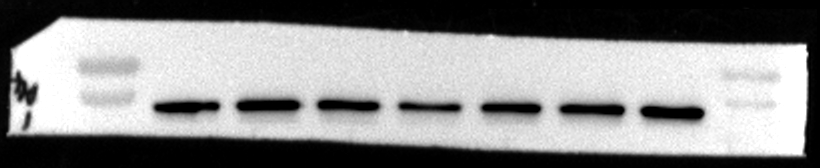


Fig. 6B

pERK
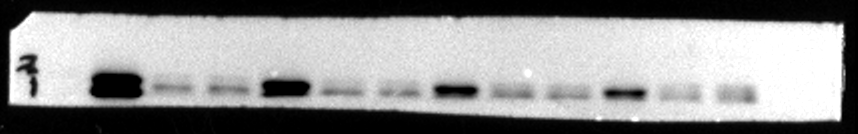


ERK
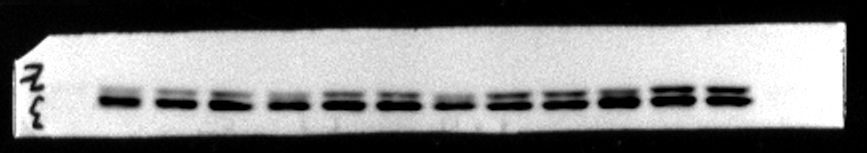


pAKT
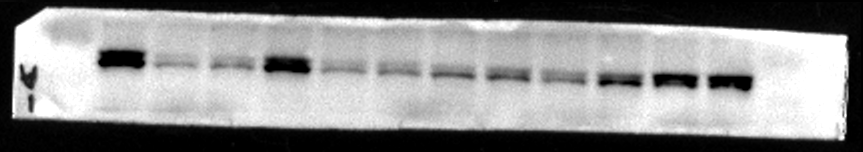


AKT
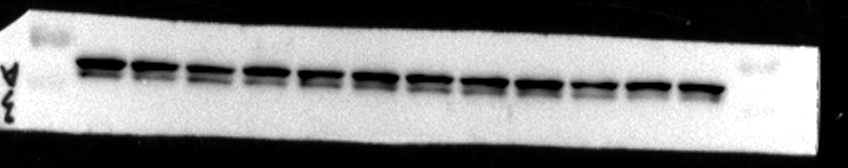


CyclinD1
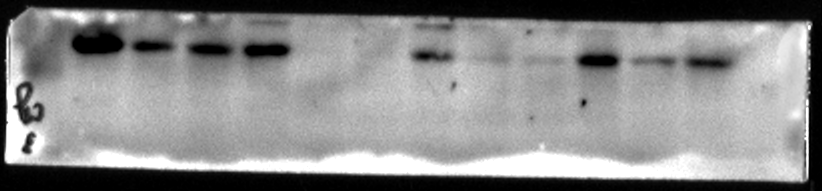


Cdk4
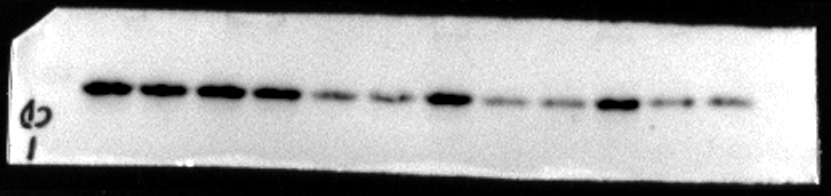


Tubulin
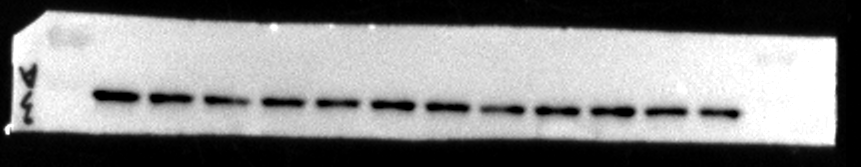


Fig. 6C

pERK
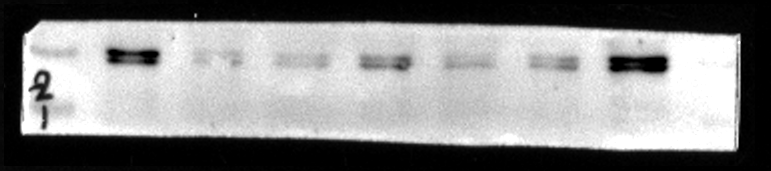


ERK
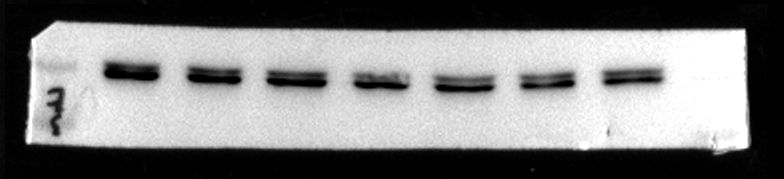


pAKT
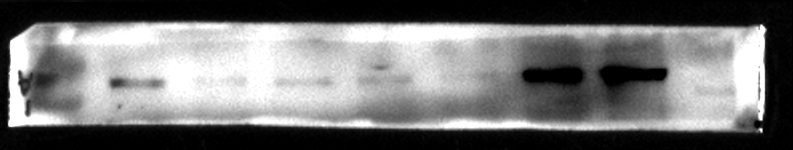


AKT
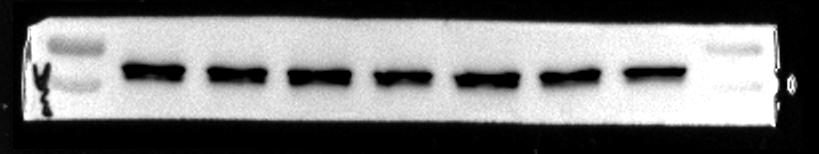


cCasp-7
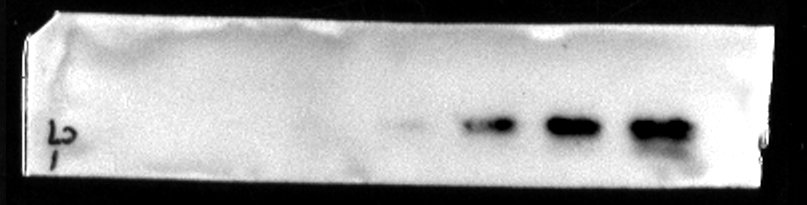


cCasp-3
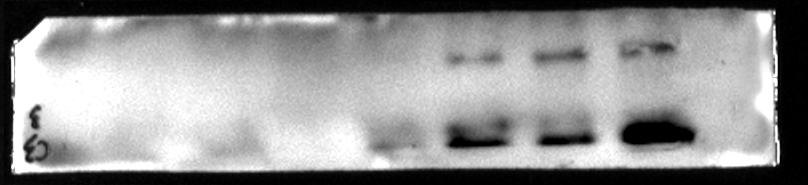


Tubulin
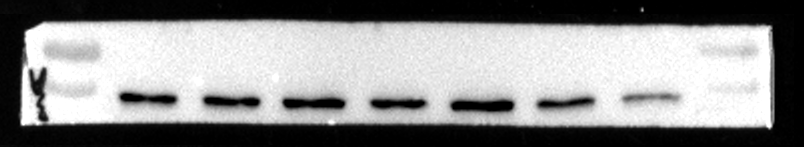


Fig. 6D

KRAS G12D
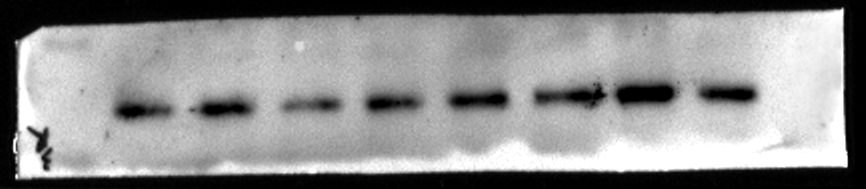


pERK
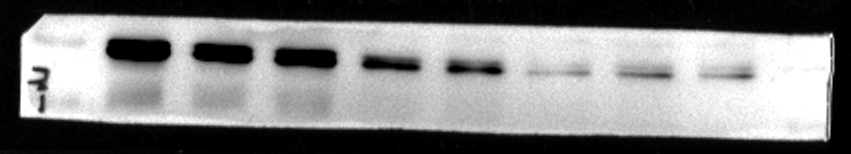


ERK
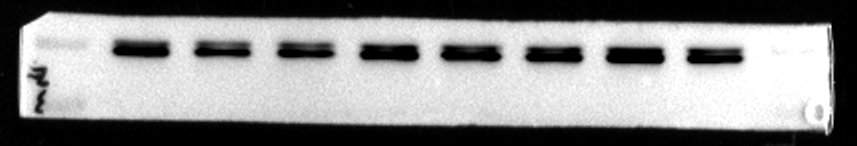


pSHP2
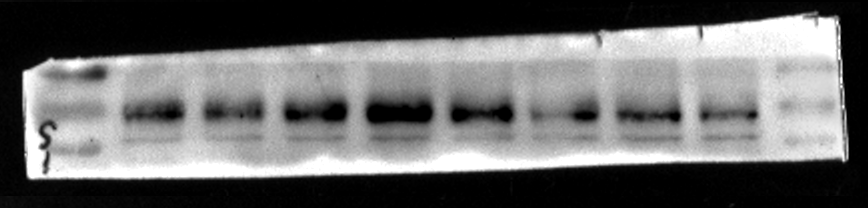


SHP2
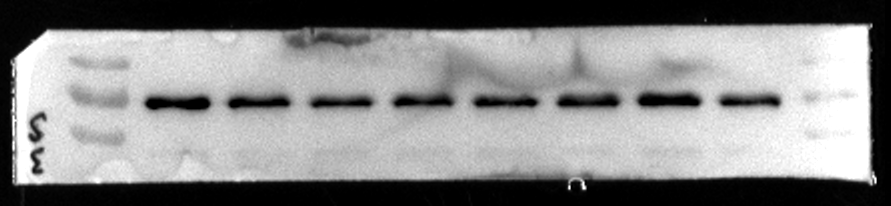


Tubulin
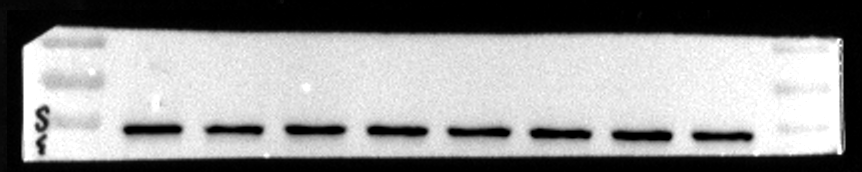

Supplement: Supplementary file 1 [file Supplementaryfile1.docx]
